# A Case of Reverse Takotsubo Cardiomyopathy After Administering a Local Anesthetic Containing Epinephrine

**DOI:** 10.7759/cureus.57476

**Published:** 2024-04-02

**Authors:** Emma R Marshall, Kurian T Maliel, Kathryn M Burtson

**Affiliations:** 1 Internal Medicine, Wright State University Boonshoft School of Medicine, Dayton, USA; 2 Internal Medicine, Wright Patterson Medical Center, Dayton, USA; 3 Cardiology, Wright Patterson Medical Center, Dayton, USA

**Keywords:** st-segment changes, active-duty military personnel, elevated troponin, epinephrine injection, takotsubo cardiomyopathy (tc)

## Abstract

Takotsubo cardiomyopathy (TCM) is a rare syndrome characterized by acute and transient distinctive wall motion abnormalities accompanied by other defined objective findings. There are many variants of TCM, including the reverse (or basal) subtype. While the pathogenesis is not fully understood, both endogenous and exogenous catecholamines have been implicated.

This case report describes a 30-year-old active-duty military female who developed reverse TCM immediately following local anesthetic with epinephrine administration in preparation for an elective septorhinoplasty. She developed electrocardiogram (ECG) changes, temporary hemodynamic instability, and cardiac troponin elevation. Transthoracic echocardiogram (TTE) demonstrated significantly reduced systolic and diastolic function, with akinesis of the basal segments and normal wall motion of the apical segments, consistent with a reverse Takotsubo pattern. Coronary computed tomography (CT) angiography showed normal coronary arteries. Repeat TTE was performed two days after the initial event and showed near-complete resolution of the wall motion abnormalities. Fourteen days later, TTE showed normalization of cardiac function.

While there is a favorable prognosis for most patients with this diagnosis, there does remain the potential for significant adverse outcomes, risk of recurrence, and a non-negligible mortality rate. It is widely known that physical and emotional triggers can precipitate TCM through the release of catecholamines. This case, in addition to numerous other case reports, provides further documentation and support that exogenous epinephrine administration is also associated with the development of TCM. Clinicians should consider the diagnosis of Takotsubo cardiomyopathy if hemodynamic or ECG changes arise following epinephrine administration.

## Introduction

Takotsubo cardiomyopathy (TCM) is a rare syndrome characterized by acute and often transient regional wall motion abnormalities extending beyond a single epicardial coronary artery distribution, electrocardiogram (ECG) changes, and cardiac enzyme elevation [1,2]. Importantly, this diagnosis also requires the absence of obstructive coronary disease or other coronary artery abnormalities as the underlying etiology. The revised Mayo Clinic criteria also include the absence of myocarditis or pheochromocytoma. The incidence of TCM is estimated to be 15 to 30 cases per 100,000 per year or 2% of all troponin-positive patients presenting with suspected acute coronary syndrome (ACS) [2]. Several variants have been identified based on the distinctive wall motion abnormalities present, including apical, basal (or reverse), mid-ventricular, and rarer variants. Of all patients diagnosed with TCM, the proportion presenting with the reverse variant ranges from 1-23% [1,2].

While the pathogenesis of Takotsubo cardiomyopathy is not fully understood, both endogenous and exogenous catecholamines have been implicated [3]. It is widely known and accepted that stressful experiences, whether psychological or physical, have been demonstrated to cause TCM, also known as “Broken Heart Syndrome” or “Stress Cardiomyopathy” [2,4]. This is thought to be due to the release of endogenous catecholamines and other neuropeptides during stress, which affect the myocardium through various proposed mechanisms [5,6]. Additionally, numerous case reports have documented the association of exogenous catecholamines in the development of TCM. Below, a case is described of reverse Takotsubo cardiomyopathy in an active-duty military female that developed after local anesthetic and epinephrine administration. This article was previously presented as a meeting abstract at the 2023 American College of Physicians Tri-Service Chapters Annual Scientific Meeting on November 8, 2023.

## Case presentation

A 30-year-old active-duty female with a history of anxiety presented to her Military Treatment Facility for an elective septorhinoplasty to be performed by her otolaryngologist. General endotracheal anesthesia was initiated without complication; medications administered intravenously included propofol, rocuronium, midazolam, hydromorphone, acetaminophen, and clindamycin. Immediately following the injection of local anesthetic (9 mL of 1% lidocaine with epinephrine 1:50,000), a wide complex tachycardia was observed on the cardiac monitor for approximately 30 seconds, accompanied by significant hypertension. This was immediately followed by sustained and diffuse ST segment depressions, in addition to hypotension requiring temporary vasopressor support with phenylephrine.

The on-call cardiologist was notified, and an emergent transthoracic echocardiogram (TTE) was performed (Videos 1-5). This TTE showed significantly reduced systolic function with a left ventricular ejection fraction (LVEF) of 35% using biplane Simpson’s method and impaired diastolic function. Concerning regional wall motion abnormalities were also noted; the basal segments appeared akinetic while the apical wall motion was normal, consistent with a reverse Takotsubo cardiomyopathy pattern. Other findings on this TTE included moderate tricuspid regurgitation and an elevated estimated pulmonary artery systolic pressure (PASP) of 64 mmHg. There was no other significant valvular disease present. The procedure was halted, vitals soon stabilized without intravenous medications, and the patient was admitted for further evaluation.

**Video 1:** Reverse Takotsubo cardiomyopathy (apical two-chamber view) An apical two-chamber view on transthoracic echocardiogram demonstrating preserved wall motion at the apex with hypokinesis of the basal/inferior wall segments.

**Video 2:** Reverse Takotsubo cardiomyopathy (apical four-chamber view) An apical four-chamber view on transthoracic echocardiogram demonstrating hypokinesis of the basal/inferior wall segments, with preserved wall motion of the apical wall segments (top of the screen).

**Video 3:** Reverse Takotsubo cardiomyopathy (parasternal short axis view) Parasternal short axis view on transthoracic echocardiogram with contrast agent administration demonstrating near-akinesis of the left ventricular basal wall segments.

**Video 4:** Reverse Takotsubo cardiomyopathy (parasternal short axis view at the level of apex) Parasternal short axis view at the level of the apex on transthoracic echocardiogram demonstrating normal apical wall motion, revealing a stark contrast from the impaired systolic function of the basal wall segments.

**Video 5:** Reverse Takotsubo cardiomyopathy (parasternal long axis view) Parasternal long axis view on transthoracic echocardiogram demonstrating significantly impaired left ventricular systolic and diastolic function, with basal wall segment akinesis noted.

A detailed history was obtained from the patient upon her emergence from anesthesia. She denied any particularly stressful events before the procedure. She also denied previous or current cardiopulmonary symptoms. The physical exam, including cardiovascular and pulmonary exams, was unremarkable. Additional objective data was obtained upon admission. Labs were significant for an elevated and rising Troponin I (0.129 ng/mL, subsequently peaking at 1.52 ng/mL four hours later) and a moderate leukocytosis (white blood cells 20.8 x 10^9^/L). N-terminal prohormone of brain natriuretic peptide (NT-proBNP) was non-elevated at 48 pg/mL. Thyroid function tests were normal. Coronary computed tomography (CT) angiography showed normal coronary arteries with no evidence of anomalous coronary arteries, spontaneous coronary artery dissection, or atherosclerosis. The ST segment depressions resolved soon after the procedure, but diffuse T wave inversions were noted to develop the following day. The patient remained asymptomatic, and troponin continued to trend down after the initial peak.

Repeat TTE two days after the procedure showed near-complete resolution of the previously observed wall motion abnormalities, with normal systolic and diastolic function (LVEF estimated at 55%). She was discharged home with a cardiac event monitor for 14 days, which showed resolution of T wave inversions and no further arrhythmias. She was started on low-dose metoprolol succinate, but unfortunately did not tolerate the medication due to side effects, and this was discontinued shortly after initiation. Additional TTE 14 days later demonstrated complete normalization of cardiac function (LVEF estimated at 58%), as demonstrated in Videos 6-9.

**Video 6:** TTE on day 14 (apical two-chamber view) Apical two-chamber view on transthoracic echocardiogram (TTE) demonstrating improved contractility of the basal wall segments, now in unison with the apical wall segments, resulting in the normalization of left ventricular systolic and diastolic function.

**Video 7:** TTE on day 14 (apical four-chamber view) Apical four-chamber view on transthoracic echocardiogram (TTE) demonstrating improved contractility of the basal wall segments, now in unison with the apical wall segments, resulting in the normalization of left ventricular systolic and diastolic function.

**Video 8:** TTE on day 14 (parasternal short axis view) Parasternal short axis view on transthoracic echocardiogram (TTE) demonstrating improvement of basal segment wall motion with normalization of left ventricular systolic and diastolic function.

**Video 9:** TTE on day 14 (parasternal long axis view) Parasternal long axis view on transthoracic echocardiogram (TTE) demonstrating improvement in basal segment wall motion with normalization of left ventricular systolic and diastolic function.

After discharge, she was appropriately restricted from fitness and her military duty. She had close and frequent follow-ups with her cardiologist and primary care provider. She gradually returned to exercise and was cleared to return to full duty as a military service member.

## Discussion

Diagnosis of Takotsubo cardiomyopathy requires a high index of suspicion and multimodality testing. As described above, our patient demonstrated the characteristic findings of reverse Takotsubo cardiomyopathy. In a young female with no significant personal or family medical history, etiologies such as spontaneous coronary artery dissection and anomalous coronary arteries must be considered with an initial presentation concerning ACS [7,8]. Coronary CT angiography demonstrated normal coronary arteries in our patient, ruling out coronary pathology.

Our patient’s demographic as a younger adult female is unusual for the development of Takotsubo cardiomyopathy; more than 90% of cases occur in postmenopausal women, assumed to be related to increased psychological stressors and estrogen deficiency in this population [1,2]. While our patient did not have a distinct psychological stressor acting as a trigger, she did have an underlying pre-existing mental health condition (generalized anxiety), which is an identified risk factor for the development of this diagnosis [2,4].

Fortunately, all variants of Takotsubo cardiomyopathy have a generally favorable prognosis, with more than 90% achieving complete recovery in one to two months [4]. However, there remains the potential for both short- and long-term adverse outcomes, including acute congestive heart failure, cardiogenic shock, arrhythmias, left ventricular outflow tract obstruction, mitral regurgitation, thrombus formation, intramyocardial hemorrhage and rupture, and death, with an estimated mortality rate of 5% [2]. The outcomes and recurrence rate of reverse TCM are similar to those of the classic apical variant of TCM [1]. Although recurrence is relatively rare, relapses have been observed in various studies. The reported prevalence of relapse ranges from 1.8% to 10% [4]. One study demonstrated a relapse rate of 1.8% per year; another study with 519 patients demonstrated that TCM recurred in 7.5% of patients over 5.2 years of follow-up; one meta-analysis involving 1664 patients reported a 5% relapse after six years [5].

It is well-recognized that physical and emotional stressors can precipitate any variant of Takotsubo cardiomyopathy through the release and action of catecholamines. Our case, in addition to numerous preceding care reports, further documents the association of exogenous epinephrine administration with the development of a variant of Takotsubo cardiomyopathy [9-12]. In one systematic review and meta-analysis, routes of epinephrine administration that have been documented include topical, local irrigation, subcutaneous, submucosal, intramuscular, and intravenous, with doses ranging from 0.04 mg to 5.0 mg [3,4]. In a case report published by Yamamoto et al. in 2021, the dose of epinephrine that was noted to precipitate TCM was as small as 0.015 mg [13]. Our case provides additional documentation that even very low doses of epinephrine (approximately 0.02 mg) can induce TCM. These cases serve as a reminder to be cautious upon administration of local anesthetics with epinephrine, particularly in areas where blood flow is abundant, and there is a risk of accidental intravascular injection.

## Conclusions

This case report presents a younger adult female who developed the rarer reverse subtype of Takotsubo cardiomyopathy after a local anesthetic containing epinephrine was injected into the nasal mucosa. When making this diagnosis, it is necessary to exclude other potential etiologies, particularly obstructive coronary artery disease or, in a younger patient, anomalous coronary arteries or spontaneous dissection. While it is well known that severe physical and psychological stress can cause this cardiomyopathy, this case report, among others, suggests that exogenous epinephrine administration is also associated with this diagnosis. Clinicians should consider Takotsubo cardiomyopathy, including its many variants, if there is evidence of hemodynamic instability or ECG changes following epinephrine administration of any route.
